# Sphingosine-1-Phosphate Receptor-1 Selective Agonist Enhances Collateral Growth and Protects against Subsequent Stroke

**DOI:** 10.1371/journal.pone.0138029

**Published:** 2015-09-14

**Authors:** Masahiko Ichijo, Satoru Ishibashi, Fuying Li, Daishi Yui, Kazunori Miki, Hidehiro Mizusawa, Takanori Yokota

**Affiliations:** 1 Department of Neurology and Neurological Science, Tokyo Medical and Dental University, Tokyo, Japan; 2 Department of Endovascular Surgery, Tokyo Medical and Dental University, Tokyo, Japan; 3 Department of Neurology, National Center of Neurology and Psychiatry, Tokyo, Japan; 4 The Center for Brain Integration Research, Tokyo Medical and Dental University, Tokyo, Japan; University of Münster, GERMANY

## Abstract

**Background and Purpose:**

Collateral growth after acute occlusion of an intracranial artery is triggered by increasing shear stress in preexisting collateral pathways. Recently, sphingosine-1-phosphate receptor-1 (S1PR1) on endothelial cells was reported to be essential in sensing fluid shear stress. Here, we evaluated the expression of S1PR1 in the hypoperfused mouse brain and investigated the effect of a selective S1PR1 agonist on leptomeningeal collateral growth and subsequent ischemic damage after focal ischemia.

**Methods:**

In C57Bl/6 mice (n = 133) subjected to unilateral common carotid occlusion (CCAO) and sham surgery. The first series examined the time course of collateral growth, cell proliferation, and S1PR1 expression in the leptomeningeal arteries after CCAO. The second series examined the relationship between pharmacological regulation of S1PR1 and collateral growth of leptomeningeal anastomoses. Animals were randomly assigned to one of the following groups: LtCCAO and daily intraperitoneal (ip) injection for 7 days of an S1PR1 selective agonist (SEW2871, 5 mg/kg/day); sham surgery and daily ip injection for 7 days of SEW2871 after surgery; LtCCAO and daily ip injection for 7 days of SEW2871 and an S1PR1 inverse agonist (VPC23019, 0.5 mg/kg); LtCCAO and daily ip injection of DMSO for 7 days after surgery; and sham surgery and daily ip injection of DMSO for 7 days. Leptomeningeal anastomoses were visualized 14 days after LtCCAO by latex perfusion method, and a set of animals underwent subsequent permanent middle cerebral artery occlusion (pMCAO) 7days after the treatment termination. Neurological functions 1hour, 1, 4, and 7days and infarction volume 7days after pMCAO were evaluated.

**Results:**

In parallel with the increase in S1PR1 mRNA levels, S1PR1 expression colocalized with endothelial cell markers in the leptomeningeal arteries, increased markedly on the side of the CCAO, and peaked 7 days after CCAO. Mitotic cell numbers in the leptomeningeal arteries increased after CCAO. Administration of the S1PR1 selective agonist significantly increased cerebral blood flow (CBF) and the diameter of leptomeningeal collateral vessels (42.9 ± 2.6 μm) compared with the controls (27.6 ± 5.7 μm; *P* < 0.01). S1PR1 inverse agonist administration diminished the effect of the S1PR1 agonist (*P* < 0.001). After pMCAO, S1PR1 agonist pretreated animals showed significantly smaller infarct volume (17.5% ± 4.0% vs. 7.7% ± 4.0%, *P* < 0.01) and better functional recovery than vehicle-treated controls.

**Conclusions:**

These results suggest that S1PR1 is one of the principal regulators of leptomeningeal collateral recruitment at the site of increased shear stress and provide evidence that an S1PR1 selective agonist has a role in promoting collateral growth and preventing of ischemic damage and neurological dysfunction after subsequent stroke in patients with intracranial major artery stenosis or occlusion.

## Introduction

Proximal intracranial arterial occlusion is associated with poor functional outcome, and the salvage of brain tissue at risk of infarction is of great interest [1]. In the acute phase of proximal middle cerebral artery (MCA) occlusion, primary collateral circulation is occasionally established via leptomeningeal anastomoses from the anterior cerebral artery (ACA) and posterior cerebral artery [2]. This collateral artery growth response varies markedly among individuals, and the presence of good collateral circulation is correlated with smaller infarction volume and better long-term neurological outcome in patients with acute ischemic stroke caused by proximal MCA occlusion [3, 4]. Stimulation of collateral artery growth may be a potential therapeutic target in ischemic stroke. The process of collateral growth is triggered by an increase in shear stress in the preexisting collateral pathways [5, 6]. Complexes of platelet endothelial cell adhesion molecule-1, vascular endothelial cell cadherin, vascular endothelial growth factor receptor 2, and G-protein-coupled receptors are known to act as mechanosensors on endothelial cells directly exposed to shear stress in the vasculature [5, 7].

Sphingosine-1-phosphate receptor-1 (S1PR1) is one of the G-protein-coupled receptors identified as essential for vascular development [8], endothelial cell proliferation [9], angiogenesis regulation [10], and stabilization of the primary vascular network [11]. Recently, it was reported that S1PR1 on endothelial cells was essential for sensing fluid shear stress *in vitro*, and that S1PR1 expression increased primarily in regions of the aorta exposed to high shear stress *in vivo* [11]. In response to shear stress, S1PR1 acts to transduce intracellular signals, including those of extracellular signal-regulated kinase (ERK) 1/2, protein kinase B (Akt), and endothelial nitric oxide synthase (eNOS) phosphorylation in endothelial cells [11]. These signaling pathways are essential for cell proliferation and vasodilation [8, 9, 11, 12]. However, the expression and function of S1PR1 in the leptomeningeal arteries of the ischemic brain are not well characterized. Here, we evaluated S1PR1 expression in the chronic hypoperfused mouse brain. We tested the hypothesis that activation of S1PR1 by means of an S1PR1-selective agonist would enhance adaptive collateral growth in a C57B1/6 mouse model of unilateral common carotid artery (CCA) occlusion (CCAO), in which shear stress stimulation of leptomeningeal anastomosis has been reported [13–15]. We also assessed the effect of the S1PR1 agonist on subsequent ischemic damage after focal ischemia.

## Materials and Methods

### Animals and Drug Administration

The study was carried out in strict accordance with the National Institutes of Health guidelines for the care and use of animals in research. All animal experiments were approved by the Institutional Animal Care and Use Committee of Tokyo Medical and Dental University (Permit Number: 0150068A). All surgery was performed under anesthesia, and every effort was made to minimize suffering. We used male C57Bl/6 mice (Sankyo Laboratory Animal Center, Tokyo, Japan) aged 10 to 14 weeks (23 to 30 g). Three series of experiments were performed. A total 119 mice were randomized and following procedures were performed in blind fashion. We excluded some mice from further analysis after randomization, according to the following criteria: (a) Severe weight loss of more than 20% of the initial body weight (b) Insufficient reduction in ipsilateral CBF less than 20% of pre-surgery CBF 1 hour after surgery for the animals divided to LtCCAO group, (c) Insufficient reduction in ipsilateral CBF less than 50% of pre-surgery CBF 1 hour after pMCAO surgery for third series of the experiment. The number of included animals is shown in S1 Table. An investigator blinded with respect to the treatment group performed all assessments.

**Fig 1:**
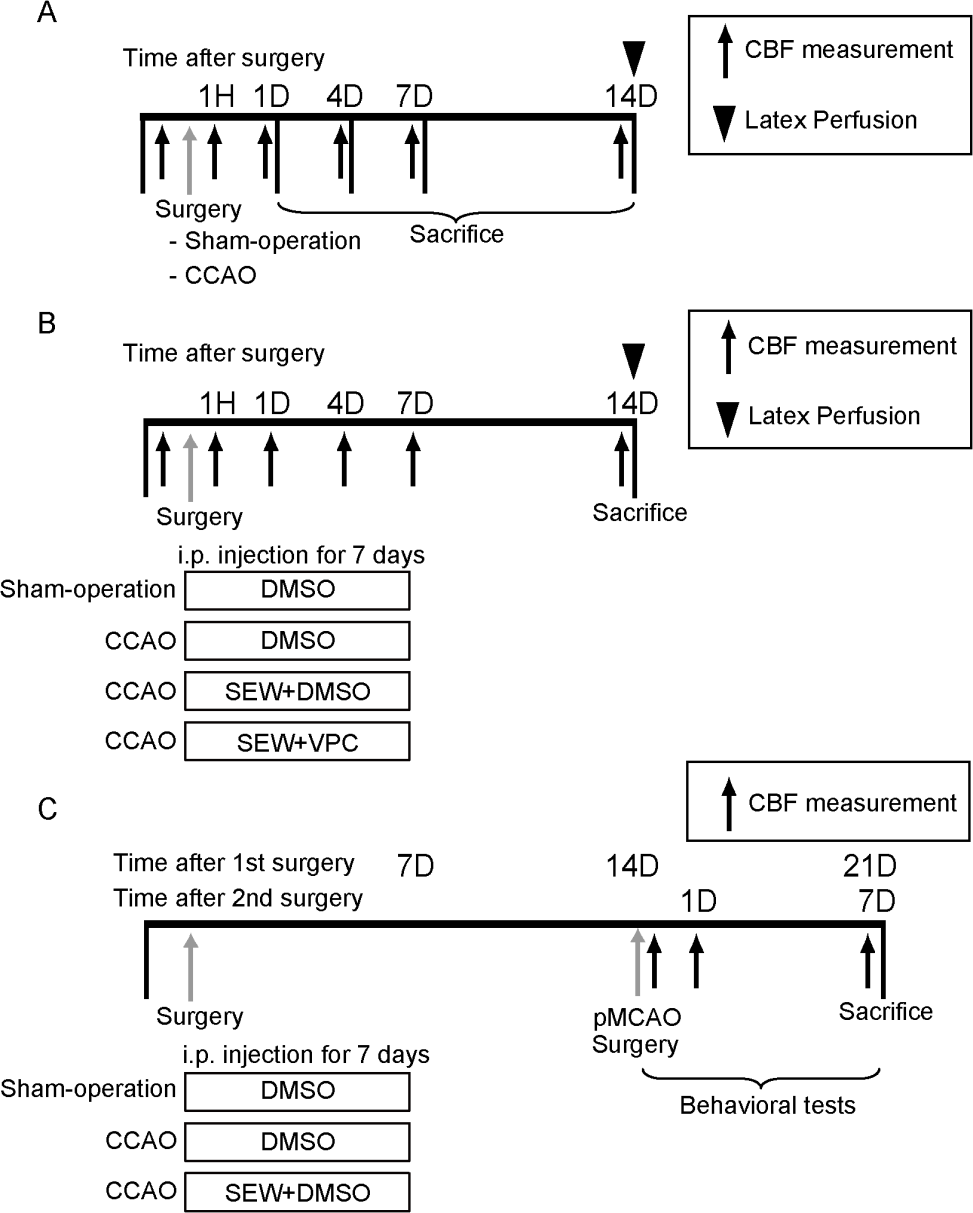
Ischemic surgery and treatment schedule. In the protocol I, animals were euthanized 1, 4, 7, or 14 days after surgery (A). In protocol II (B) and III (C). SEW, SEW+VPC, or control vehicle (DMSO) were administered intraperitoneally for 7 days after surgery.

The first series examined the time course of collateral growth, cell proliferation in the leptomeningeal arteries (Fig 1A), and S1PR1 expression in the leptomeningeal arteries after left common carotid artery occlusion (LtCCAO) surgery. Fourteen mice randomly assigned to sham surgery (n = 7) and LtCCAO surgery (n = 7), and leptomeningeal anastomoses were visualized and assessed by latex perfusion method. To assess the time course of histological changes and quantitative reverse transcription PCR (qRT-PCR) analysis, 44 mice in this series randomly divided to sham surgery (n = 8) and LtCCAO surgery (n = 36). LtCCAO animals randomly assigned to 4 subgroups that were euthanized 1 day (n = 9), 4 days (n = 9), 7 days (n = 9), and 14 days (n = 9) after CCAO surgery. The second series examined the relationship between pharmacological regulation of S1PR1 and collateral growth of leptomeningeal anastomoses. Fifty seven mice were randomly assigned to one of the following groups: LtCCAO and daily intraperitoneal (ip) injection for 7 days from 1hour after surgery of an S1PR1 selective agonist (SEW2871, Cayman Chemical Company, Ann Arbor, MI, 5 mg/kg/day) diluted with 0.3 mL dimethyl sulfoxide (DMSO) (SEW group, n = 15); sham surgery and daily ip injection for 7 days after surgery of SEW2871 (SEW without CCAO group, n = 6); LtCCAO and daily ip injection for 7 days after surgery of SEW2871 and an S1PR1 inverse agonist (VPC23019, Avanti Polar Lipids, Alabaster, AL, 0.5 mg/kg) diluted with 0.3 mL DMSO (SEW+VPC group, n = 8); LtCCAO and daily ip injection of DMSO (0.3 mL/day) for 7 days after surgery (vehicle group, n = 15); and no occlusion and daily ip injection of DMSO (0.3 mL/day) for 7 days after sham surgery (sham group, n = 13) (Fig 1B). Each treatment group randomly assigned to undergo latex perfusion analysis (n = 31) or histological analysis (n = 26). Latex perfusion analysis was performed 14 days after LtCCAO. Animals for histological analysis randomly divided to 2 subgroups that were euthanized 7 days and 14 days after LtCCAO. The third series examined the effect of treatment to the infarction volume or neurological outcome. Eighteen mice were divided at random into sham (n = 6), vehicle (n = 6), and SEW group (n = 6), underwent permanent middle cerebral artery occlusion (pMCAO) 1 week after the treatment termination (Fig 1C).

### Common Carotid Artery Occlusion

Mice were anesthetized with 2.5% isoflurane and a mixture of 30% oxygen and 70% nitrous oxide via a facemask. Physical condition including body temperature and breathing were constantly monitored during the surgery and 4 h thereafter. Body temperature was monitored with a deep rectal thermometer and maintained at 36.5±0.5°C with a heating pad. The left CCA was exposed through a midline incision and ligated with a silk suture as previously reported [13–15]. Sham surgery was performed by using the same process but without ligation.

### Middle Cerebral Artery Occlusion Subsequent to Common Carotid Artery Occlusion

Mice were anesthetized with 2.5% isoflurane. Body temperature was monitored with a deep rectal thermometer and maintained at 36.5±0.5°C with a heating pad during surgery and for 4 h thereafter. Mice were placed in lateral recumbency and a vertical skin incision was made at the midpoint between the left orbit and the external auditory canal. After reflection of the temporal muscle and soft tissue, the left MCA was visualized through the skull. A small burr hole (2mm in diameter) was made with a microdrill through the outer surface of the skull at the junction between the medial wall and the roof of the inferotemporal fossa at the skull base to expose the left middle cerebral artery (MCA) at the point between the inferior cerebral vein and the lateral olfactory tract. The left MCA was occluded permanently by using a microbipolar electrocoagulator (ICC 200; ERBE USA Inc., Atlanta, GA).

### Measurement of Superficial Cerebral Blood Flow (CBF) with Laser-Doppler Flowmetry

A laser Doppler flow meter (TBF-LN1; Unique Medical Company, Tokyo, Japan) and a laser Doppler probe (Type-CS; Advance Co, Inc, Tokyo, Japan) were used to monitor relative changes in blood flow. The animals were anesthetized with 2% isoflurane and the skin overlying the skull was reflected. Two silicone rings (outer diameter 5.0 mm, inner diameter 3.0 mm, width 1.0 mm) for a laser-Doppler flowmetry probe were fixed to the skull side by side; the center of each ring was located 1.0 mm posterior and 2.5 mm lateral to the bregma (one ring on each side) with binding material and dental resin. CBF was recorded by placing a 3.0-mm straight probe through one of the guide rings, depending on the position chosen for the measurement. The mice were placed on a heating pad to maintain a constant body temperature of 36.5±0.5°C for the entire procedure. Baseline CBF values were recorded just before surgery and 1 h and 1, 4, 7, and 14 days after surgery. CBF values were expressed as percentages of the pre-surgery value.

### Latex Perfusion

Leptomeningeal anastomoses were visualized 14 days after LtCCAO as described previously [14, 15]. Under deep anesthesia, the auricle of the right atrium was incised to allow venous outflow; the left ventricle of the heart was cannulated via the left ventricular apex, and 3 mL saline was injected. Immediately after the saline injection, 0.5 mL white latex compound (Product No. 563;Chicago Latex Products Inc, Crystal Lake, IL) mixed with 50 μL/mL carbon black ink (Bokusai; Fueki Inc, Tokyo, Japan) diluted 10:1 with saline was infused at an injection pressure of 150 mmHg. The brain was removed carefully and fixed in Zamboni solution (2% paraformaldehyde and 0.2% picric acid) for 24 h. Photographs of the surface of the brain were taken to assess the diameter and number of leptomeningeal anastomoses in both hemispheres. Leptomeningeal anastomoses between the MCA and the ACA were located by tracking the distal branches of each artery to the point of confluence. The diameters of leptomeningeal anastomoses were measured by using NIH Image software.

### Tissue Preparation and Measurement of Total Infarct Volume

At 1, 4, 7, and 14 days after LtCCAO, 7 days after pMCAO, and 4 days after sham surgery, the animals’ tissues were fixed by transcardiac perfusion with 4% paraformaldehyde under deep anesthesia. The brains were removed, incubated in 20% sucrose, and then frozen rapidly on dry ice. The brains were then cut into 20-μm serial coronal sections from the level of the anterior pole of the caudate nucleus through the cerebral hemisphere, mounted on slides, and processed for staining. To evaluate ischemic lesions, eight serial sections, spaced 200 μm apart from bregma level +1.2 mm to –2.0 mm, were stained with cresyl violet. The areas of the infarct, ipsilateral hemisphere, and contralateral hemisphere in the eight coronal sections were measured on an image of each section by using NIH Image analysis software. The total infarct volume of the ipsilateral hemisphere (% infarction volume) was calculated as a percentage of the volume of the contralateral hemisphere.

### Immunohistochemistry and Cell Quantification

The primary antibodies used for the immunohistochemical staining were as follows: rabbit anti-rat Ki-67 monoclonal antibody (1:500; Acris, Herford, Germany) for detection of mitotic cells; rabbit anti-eNOS (phosphor S1777; 1:500; Abcam, Cambridge, UK) antibody for detection of eNOS phosphorylation (peNOS); rabbit anti-mouse α-smooth muscle actin (α-SMA; 1:500; Thermo Fisher Scientific, Fremont, CA) for detection of smooth muscle cells; rat anti-mouse CD31 antibodies (PECAM-1, 1:50, BD Pharmingen, San Diego, CA) for detection of endothelial cells; and mouse anti-sphingosine-1-phosphate receptor 1 antibody (S1PR1, 1:200, EMD Millipore, Temecula, CA) for detection of S1PR1.

Single immunostaining for Ki67 was performed to evaluate cellular proliferation in the vessels of the leptomeningeal anastomoses. Ki-67 immunostaining required antigen retrieval by heating in 10 mmol/L citrate buffer (pH 6) in a microwave oven (5×2 min, 750 W). The 20-μm-thick sections were rinsed at room temperature three times for 5 min each in PBS containing 0.1% Triton X-100 (Sigma Aldrich, St. Louis, MO). Endogenous peroxidase activity was blocked with 0.3% hydrogen peroxide, followed by preincubation with 10% normal goat serum, and then incubated overnight at 4°C with primary antibody diluted with 10% normal goat serum. After three washes in PBS, the sections were further incubated at room temperature for 1 h with biotinylated anti-rabbit immunoglobulin G (1:200; Vector Laboratories, Burlingame, CA) and then with streptavidin–biotin–horseradish peroxidase complex (Vector Laboratories) for 1 h at room temperature. Immunoreactivity was visualized with 3,3′-diaminobenzidine (Sigma Aldrich, St. Louis, MO). For double immunohistochemical staining, the 20-μm-thick sections were washed with PBS three times and blocked with 10% normal goat serum in PBS, and then incubated overnight at 4°C with the two primary antibodies diluted with 10% normal goat serum. Subsequently, sections were incubated in fluorescently labeled secondary antibodies (fluorescein isothiocyanate / rhodamine, raised in goat; EMD Millipore) for 1 h at room temperature. After washes in PBS, the sections were mounted with Vectashield (Vector Laboratories) and observed under a confocal laserscanning microscope (LSM510; Zeiss, Oberkochen, Germany).

### Cell Quantification

To estimate the total number of Ki67-positive cells and double positive area of S1PR1 / CD31 and peNOS / CD31 in the vessels of the ipsilateral leptomeningeal anastomoses, eight serial sections, spaced 200 μm apart from bregma level +1.2 mm to –2.0 mm, were collected from each mouse. Counts of Ki67-positive cells in ipsilateral leptomeningeal anastomoses are given as cells per section. The S1PR1^+^CD31^+^ and peNOS^+^CD31^+^ area as a percentage of the total CD31^+^ area in the leptomeningeal anastomoses was determined by using a confocal laser scanning microscope with a 40× objective and NIH image software.

### Quantitative reverse transcription PCR (qRT-PCR)

To evaluate the expression pattern of S1PR1, we performed quantitative reverse transcription PCR (qRT-PCR) as following methods. The temporo-parietal cotex including leptomeningeal anastomosis were dissected, immediately frozen in liquid nitrogen and homogenized in Isogen buffer (Nippon Gene) to extract RNA by manufacturer's protocol. Reverse transcription of RNA to cDNA was performed with SuperScript Reverse transcriptase (Invitrogen) using the following conditions: 25°C for 10 min, 37°C for 50 min, and 85°C for 5 min. Quantitative PCR was performed subsequently on the resulting DNA using Roche SYBR green master mix with the following primers: GAPDH: 5’-GCACAGTCAAGGCGAGAAT-3’ and 5’-GCCTTCTCCATGGTGGTGAA-3’, S1PR1: 5’-CGGTGTAGACCCAGAGTCCT-3’ and 5’-AGCTTTTCCTTGGCTGGAG-3’. Specificity of each primer set was checked by the melting curve analysis upon quantitative PCR.

### Evaluation of Neurological Function

To assess the effects of S1PR1 activation on neurological function after stroke, pMCAO animals with sham surgery (sham group), LtCCAO surgery with DMSO administration (vehicle group), and LtCCAO surgery with SEW treatment (SEW group) were subjected to a series of behavioral tests form 1 week after the treatment. Neurological deficits were assessed by using a neurological scoring system widely used in mice [16]. The neurological deficit scores were as follows: 0, normal motor function; 1, flexion of torso and contralateral forelimb when lifted by the tail; 2, circling to the contralateral side when held by the tail on a flat surface, but normal posture at rest; 3, leaning to the contralateral side at rest; 4, no spontaneous motor activity. The EBST has been used to evaluate asymmetric motor behavior in rats [17]. Mice were held by the base of the tail and elevated approximately 10 cm from the tabletop. The direction of body swing, defined as an upper body turn of >10° to either side, was recorded for 1 min during each of three trials per day. The numbers of left and right turns were counted, and the percentage of turns made to the side contralateral to the lesioned hemisphere (% right-biased body swing) was determined.

### Statistics

The independent samples Student’s t-test, two-way factorial analysis of variance (ANOVA), or one-way ANOVA with post hoc Tukey–Kramer test was used to analyze most data, as indicated in the figure legends. Repeated-measures ANOVA with the post hoc Tukey–Kramer test was used to analyze the data of behavioral tests. All values are presented as means ± s.d. *P* < 0.05 was considered to be statistically significant.

## Results

### Assessment of Change in CBF of Border Zone Area and Cell Proliferation, and Characterization of Leptomeningeal Arteries after LtCCAO

**Fig 2:**
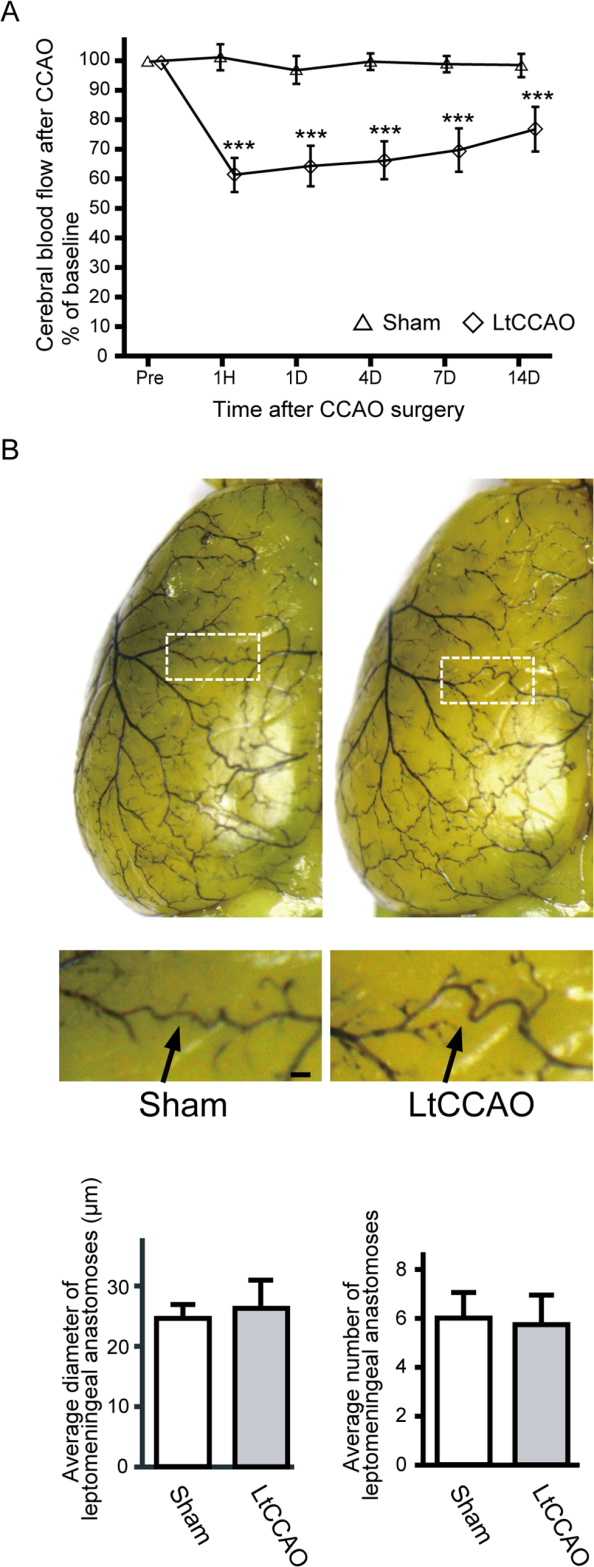
Changes in CBF and leptomeningeal anastomosis after LtCCAO. (A) Changes in CBF in the border zone between the middle cerebral artery (MCA) and anterior cerebral artery (ACA) after CCAO surgery (n = 7 for each group; ***P < 0.001 compared with sham; repeated-measures ANOVA followed by Tukey–Kramer post hoc test). (B) Representative images of superficial vessels on the ipsilateral hemisphere after LtCCAO, as assessed after latex perfusion (bars = 100 μm). Magnified images of the boxes in the upper panels are shown in the lower panels. Leptomeningeal artery between the ACA and MCA is indicated by arrows. The bar graphs indicates average diameter and average number of leptomeningeal anastomoses in the area between the ACA and MCA, as assessed after latex perfusion. CBF, cerebral blood flow; LtCCAO, left common cerebral artery occlusion.

**Fig 3:**
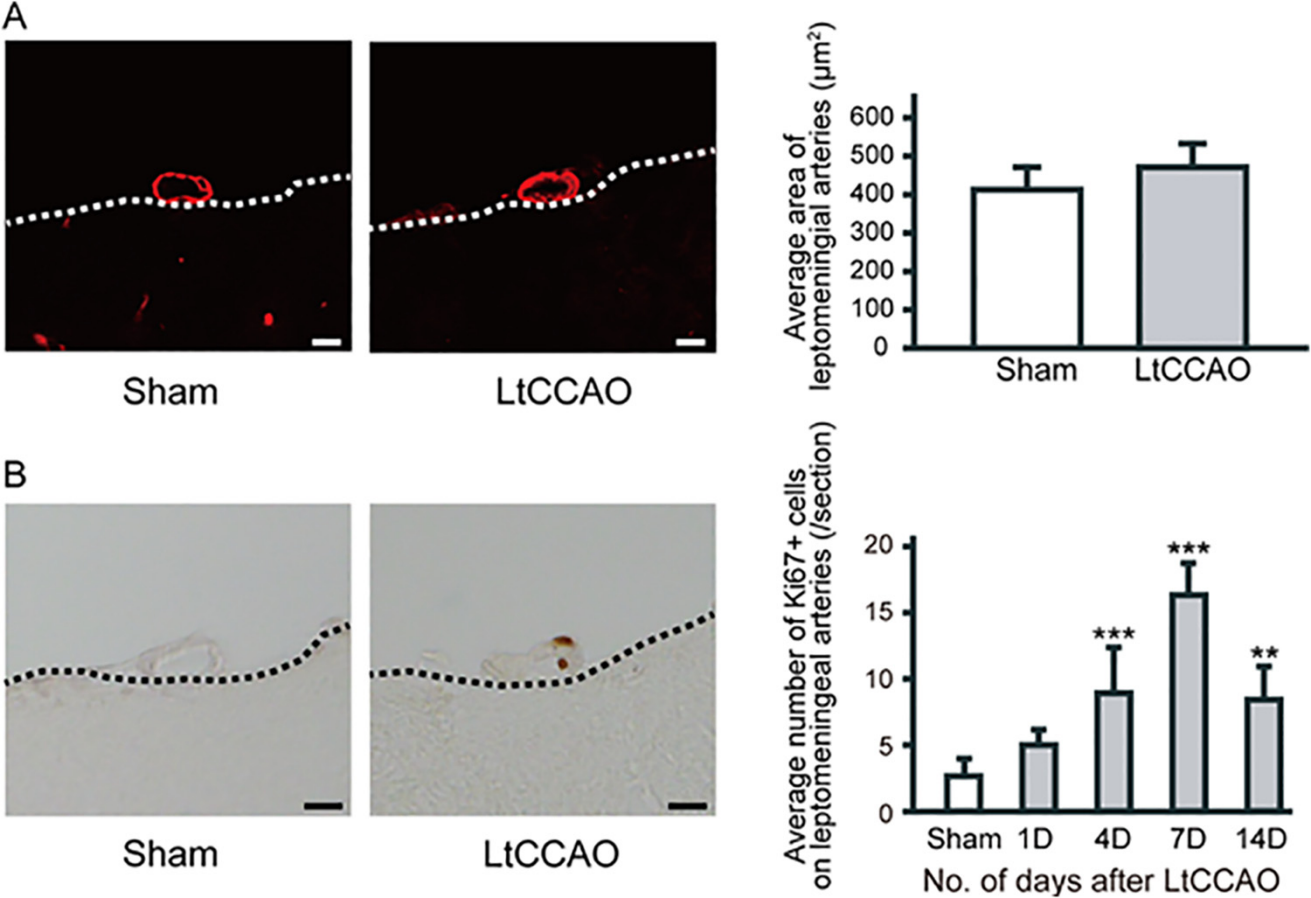
Significant increase in the number of proliferating cells in the ipsilateral leptomeningeal arteries after LtCCAO. Representative images of α-smooth muscle actin staining (A) of the ipsilateral leptomeningeal artery 2 weeks after sham surgery or LtCCAO surgery (scale bars = 10 μm). Dot line indicates the surface of cerebral cortex. There was no significant increase in the average area of ipsilateral leptomeningeal arteries after LtCCAO (B). Representative images of Ki-67 staining of ipsilateral leptomeningeal arteries (bars = 10 μm). The average number of Ki-67-positive cells in the leptomeningeal arteries (per section) after LtCCAO surgery increased until 7 days and then decreased. (n = 6 for each group; **P < 0.01, ***P < 0.001 compared with sham-operated controls; one-way ANOVA followed by Tukey–Kramer post hoc test).

Mean CBF in the border zone relative to the preoperative baseline was examined after surgery in the LtCCAO mice and compared with the CBF in sham-operated mice. No animals, irrespective of surgery or treatment, died during the study, and no animals were excluded from this experiment or analysis. CBF values after LtCCAO decreased to 61.3% ± 3.8% at 1 h, 62.0% ± 5.6% at 1 day, 67.4% ± 5.4% at 4 days, and 70.9% ± 5.5% at 7 days and were still significantly lower at 14 days (76.4% ± 5.8%) than those in sham-operated mice (Fig 2A) (group effect, F_1,12_ = 268.5, *P* < 0.001; group × trial interaction, F_5, 60_ = 70.6, *P* < 0.001). Latex perfusion clearly revealed the leptomeningeal arteries on the mice brain (Fig 2B); there was no significant difference in the number or diameter of leptomeningeal anastomoses between the sham and LtCCAO mice (Fig 2B). We quantified the area of leptomeningeal arteries in α-SMA-immunostained sections (Fig 3A). After LtCCAO, there was no significant difference in the average area of leptomeningeal arteries between the sham (421.1±50.2 μm^2^) and LtCCAO (480.2±60.2 μm^2^) mice (Fig 3A). Ki-67 immunostaining showed mitotic cells in the leptomeningeal arteries. The average number of Ki-67-positive cells (per section) in the ipsilateral leptomeningeal arteries was significantly greater at 4 days (9.0 ± 3.4, *P* < 0.001), 7 days (16.5 ± 2.3, *P* < 0.001), and 14 days (8.5 ± 2.6, *P* < 0.01) after LtCCAO than in the sham-operated mice (2.6 ± 1.4) (Fig 3B).

### Increased S1PR1 Expression on Endothelial Cells in Ipsilateral Leptomeningeal Arteries after LtCCAO

**Fig 4:**
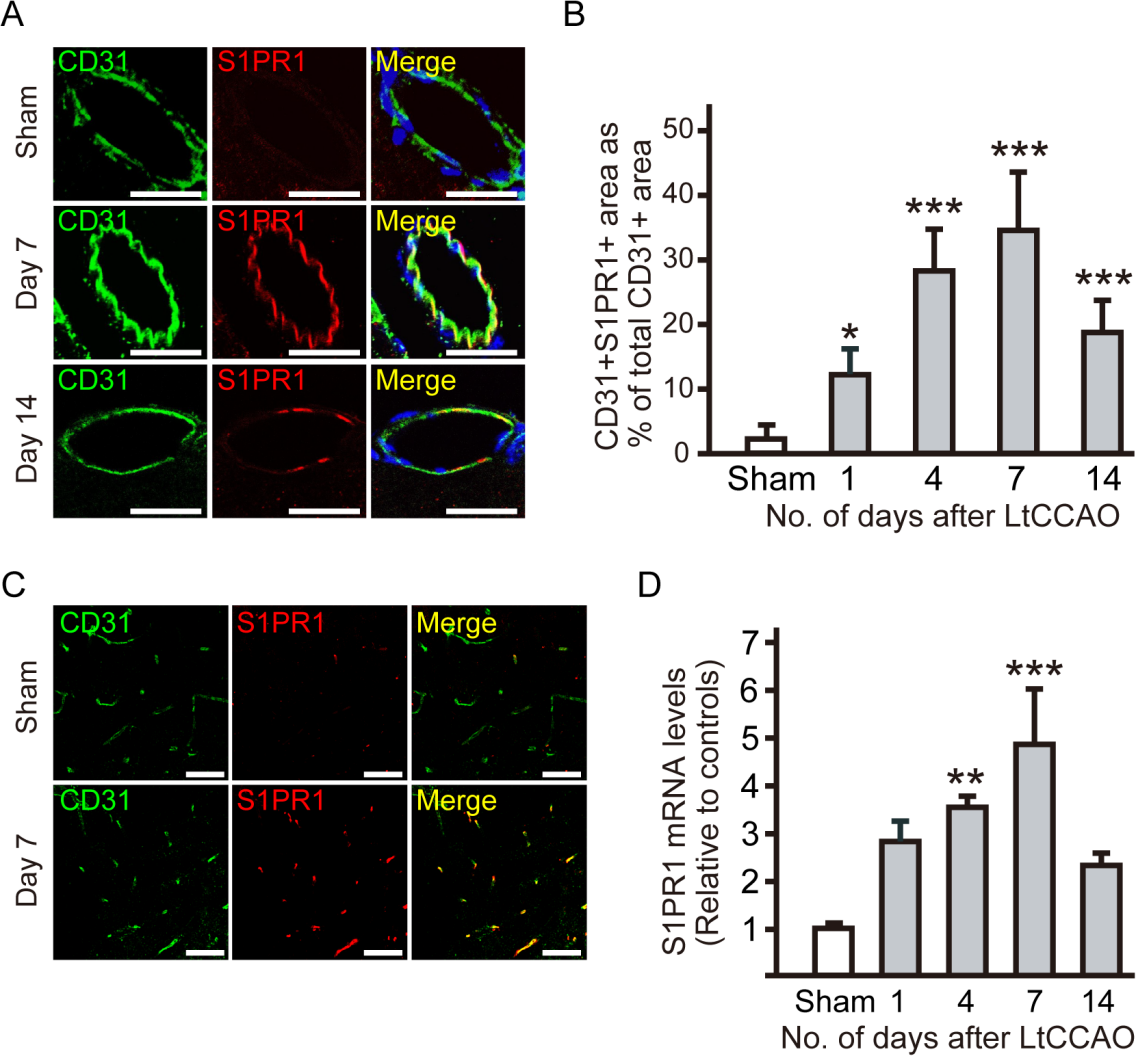
Increase in expression of sphingosine-1-phosphate receptor-1 (S1PR1) in ipsilateral leptomeningeal arteries after LtCCAO. (A) Confocal immunofluorescence double-labeling images with anti-CD31 (green) and anti-S1PR1 (red) antibodies in the ipsilateral leptomeningeal arteries 7 and 14 days after LtCCAO and in the sham-operated control. The strong S1PR1 signals (red) were detected at 7 days, but weak at 14 days after LtCCAO (bars = 10 μm). (B) Average percentage of the CD31/S1PR1 double-positive area of the total CD31 positive area after LtCCAO increased until 7 days and then decreased. (n = 6 for each group; *P < 0.05, ***P < 0.001 compared with sham-operated controls; one-way ANOVA followed by Tukey–Kramer post hoc test). (C) Confocal immunofluorescence double-labeling images with anti-CD31 (green) and anti-S1PR1 (red) antibodies in the ipsilateral parenchyma 7 days after LtCCAO and in the sham-operated control (bars = 50 μm). (D) Measurement of S1PR1 mRNA levels in ipsilateral cortex of sham-operated and ltCCAO animals. (n = 2 for sham, 3 for each CCAO group; **P < 0.01, ***P < 0.001 compared with sham-operated controls; one-way ANOVA followed by Tukey–Kramer post hoc test).

We examined the expression patterns of S1PR1 in the leptomeningeal anastomoses after LtCCAO. S1PR1 signals were observed to primarily colocalize with CD31, a marker of endothelial cells, on the ipsilateral leptomeningeal anastomoses (Fig 4A). These CD31 and S1PR1 double-positive areas as percentages of the total CD31-positive areas in the ipsilateral leptomeningeal arteries were significantly greater at 4 days (28.4% ± 6.3% per vessel), 7 days (34.5% ± 9.2% per vessel), and 14 days (19.4% ± 4.3% per vessel) after LtCCAO than in sham-operated controls (3.0% ± 1.6% per vessel, *P* < 0.001 for day4, 7, and 14), and also at 1 day (*P* < 0.05) (Fig 4B), but the percentage of these CD31 and S1PR1 double-positive areas did not increase in the contralateral leptomeningeal arteries after LtCCAO. Immunofluorescence staining showed that S1PR1 signal was apparently increased on endothelial cells in the ipsilateral cortex after LtCCAO rather than sham surgery (Fig 4C). S1PR1 mRNA level of ipsilateral cerebral cortex was significantly increased 7days after CCAO, as assessed by qRT-PCR (Fig 4D).

### Potential of S1PR1 Activation to Promote Leptomeningeal Collateral Growth under Shear Stress

**Fig 5:**
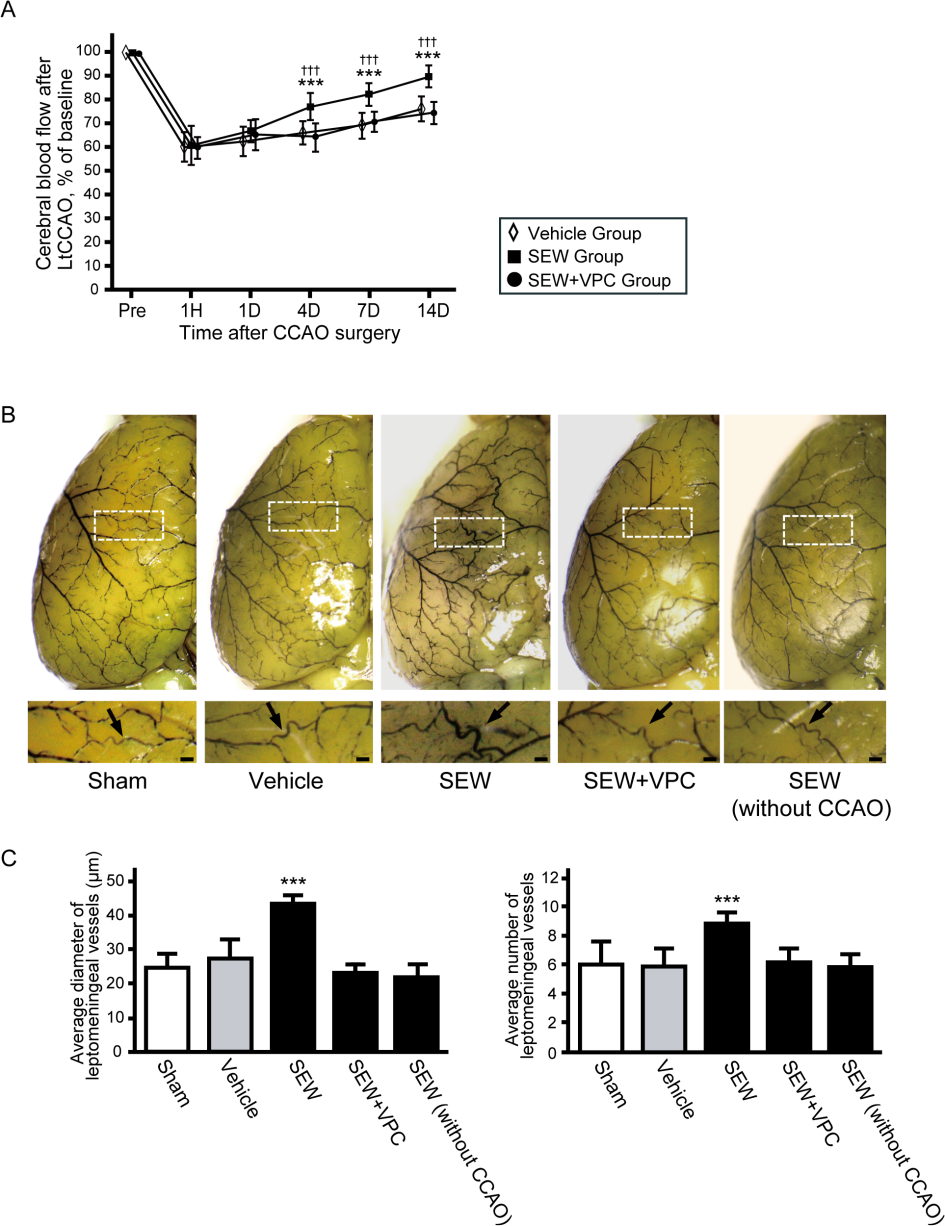
SEW2871 treatment promotes leptomeningeal collateral growth under shear stress conditions. (A) Changes in cerebral blood flow (CBF) in the border zone between the anterior cerebral artery (ACA) and middle cerebral artery (MCA) in each group (n = 11 for vehicle, 11 for SEW, and 8 for SEW+VPC; ***P < 0.001 compared with vehicle, ^†††^ *P* < 0.001 compared with SEW+VPC group; repeated-measures ANOVA followed by Tukey–Kramer post hoc test.) (B) Representative images of superficial vessels on ipsilateral hemisphere after left common carotid artery occlusion (LtCCAO), as assessed after latex perfusion. Magnified images of boxes in the upper panels are shown in the lower panels. Arrows indicate leptomeningeal anastomoses between the ACA and MCA (bars = 100 μm). (C) Average diameters and average numbers of leptomeningeal anastomoses between the ACA and MCA, as assessed after latex perfusion. (n = 7 for vehicle, 7 for SEW, 6 for SEW without CCAO, and 4 for SEW+VPC ***P < 0.001 compared with vehicle group; one-way ANOVA followed by Tukey–Kramer post hoc test). SEW, sphingosine-1-phosphate receptor-1 (S1PR1) selective agonist; VPC, S1PR1 inverse agonist.

To examine the effects of an S1PR1 selective agonist on leptomeningeal collateral growth in a mouse model of hypoperfusion, we analyzed the changes in CBF and the numbers and diameters of leptomeningeal anastomoses after LtCCAO among treatment groups (Fig 1B). No animals were excluded from this experiment or analysis. The reduction in ipsilateral CBF 1 h after LtCCAO did not differ significantly among the groups. CBF was significantly higher in the SEW group than in the vehicle group after LtCCAO at 4, 7, and 14 days after LtCCAO (group effect, F_2, 27_ = 13.3, *P* < 0.001; group × trial interaction, F_10, 46_ = 7.65, *P* < 0.001) (Fig 5A). There was no significant difference in the diameter of the vessels in the leptomeningeal anastomoses between the sham group (24.9 ± 4.0 μm), the vehicle group (27.6 ± 5.7 μm), and the SEW without CCAO group (22.3 ± 3.0 μm). However, the average area of leptomeningeal arteries (S1A Fig) and the diameter of the vessels in the leptomeningeal anastomoses in the SEW group (42.9 ± 2.6 μm) were significantly larger than those in the vehicle group (Fig 5B and 5C) (*P* < 0.001). Furthermore, the number of leptomeningeal anastomoses was significantly greater in the SEW group (8.8 ± 0.8) than in the vehicle group (Fig 5C) (5.9 ± 1.2, *P* < 0.001, per animal). The number (6.3 ± 1.0) and diameter (23.2 ± 2.3μm) of leptomeningeal anastomoses significantly decreased in SEW+VPC group, compared with SEW group (*P* < 0.001), but no significant difference between the vehicle and SEW+VPC groups (Fig 5C). Brain angioarchitecture of both sides were shown in S2A Fig. Significant increases in collateral diameter and number were observed only on the ipsilateral side (S2B Fig). CD31 and peNOS double-positive areas as percentages of the total CD31-positive areas in the ipsilateral leptomeningeal arteries 14 days after LtCCAO were significantly greater in SEW group (25.1 ± 4.2) compared with vehicle group (16.7 ± 2.8, *P* < 0.05).

There were no difference in the number of Ki-67-positive mitotic cells in the ipsilateral leptomeningeal arteries 14 days after LtCCAO between SEW group (12.3 ± 6.0) and vehicle group (8.3 ± 3.2, *P* = 0.29) (S1B Fig).

### Protective Effects of S1PR1 Activation after Subsequent pMCAO

**Fig 6:**
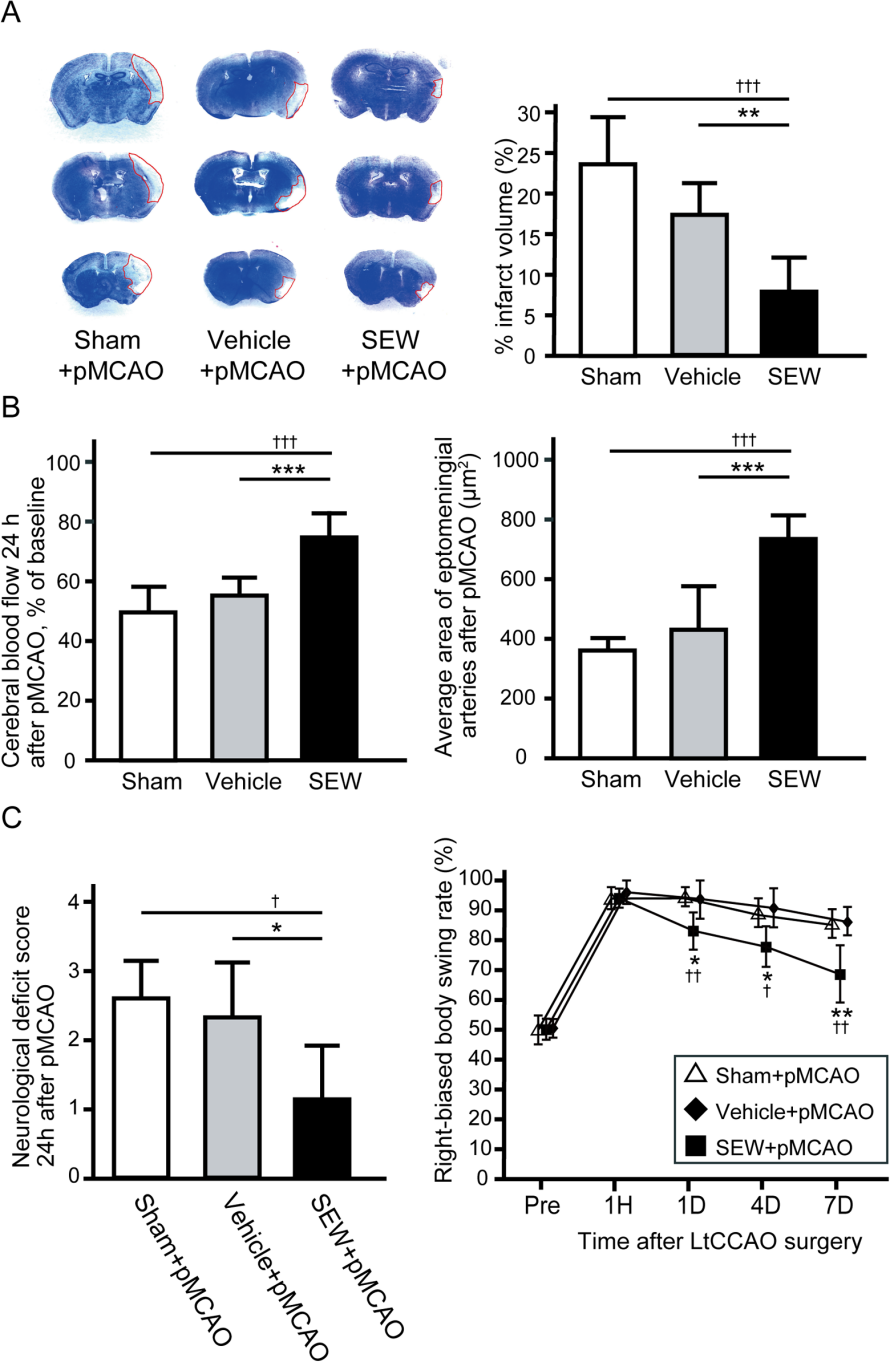
Total infarction volume reduction and improvement in neurological dysfunction with SEW treatment before pMCAO. (A) Cresyl violet staining of the brain after pMCAO in the sham group, vehicle group, and SEW group is represented. Bar graph indicates total infarct volumes measured in eight coronal sections from the sham group, vehicle group, and SEW group 1 week after pMCAO. (B) Cerebral blood flow in the border zone 24 h after pMCAO (**left**). Average areas of ipsilateral leptomeningeal arteries in the sham, vehicle, and SEW groups (**right**). (C) The neurological deficit score 24 h after pMCAO (**left**) was less severe in the SEW group than in the vehicle or sham group (n = 5 for sham, 6 for vehicle, and 6 for SEW; ^†^ *P* < 0.05, ^††^ *P* < 0.01, ^†††^ *P* < 0.001 compared with sham group, *P < 0.05, **P < 0.01, ***P < 0.001 compared with vehicle group by one-way ANOVA followed by Tukey–Kramer post hoc test). As assessed by the elevated body swing test (**right**), animals displayed more frequent turns toward the contralateral side (right) after pMCAO. Animals in the SEW group showed significantly improved recovery of right-biased body swing rate compared with those in the sham and vehicle groups (n = 5 for sham, 6 for vehicle, and 6 for SEW; ^††^ *P* < 0.01, ^†^ *P* < 0.05 compared with sham group, **P < 0.01, *P < 0.05 compared with vehicle group by repeated-measures ANOVA followed by Tukey–Kramer post hoc test). SEW, sphingosine-1-phosphate receptor-1 (S1PR1) selective agonist; pMCAO, permanent middle cerebral artery occlusion.

One mouse in sham group was excluded after randomization for insufficient CBF reduction after pMCAO surgery. The excluded mouse was humanely euthanized by pentobarbital overdose. There were no significant difference in the infarction volume 7 days after pMCAO between vehicle group and sham group (*P* = 0.07) (Fig 6A). The total infarct volume 7 days after pMCAO was significantly lower in the SEW group than in the vehicle group (7.7% ± 4.0% vs. 17.5% ± 4.0%, *P* < 0.01) (Fig 6A). CBF after pMCAO was significantly greater in the SEW group than in the vehicle group 24 hours after pMCAO (Fig 6B). Average area of ipsilateral leptomeningeal arteries 7 days after pMCAO was significantly larger in SEW group (737.6±69.6 μm^2^) than those in the vehicle group (433.8±138.8 μm^2^, *P* < 0.01) or sham group (365.1±27.8 μm^2^, *P* < 0.001) (Fig 6B), which indicate that SEW pretreatment produced persistent effect on dilating leptomenigeal arteries and improving cortical blood flow in periinfarct area after pMCAO than vehicle treatment. The neurological deficit score 1 day after pMCAO was significantly lower in the SEW group (1.17 ± 0.75) than in the vehicle group (2.33 ± 0.82, *P* < 0.05) (Fig 6C). pMCAO animals showed a strong tendency to turn toward the contralateral side. After pMCAO, SEW-treated animals partly recovered from this biased body swing, and the right-biased body swing rate was significantly lower in the SEW group than in the vehicle group at 1, 4, and 7days after pMCAO (*P* < 0.05 for 1day, 4days, and *P* < 0.01 for 7 days)(group effect, F_2, 14_ = 15.1, *P* < 0.001; group × trial interaction, F_8, 22_ = 2.3, *P* = 0.06) (Fig 6C). These results indicate that SEW pretreatment in hypoperfused mice brain could protect ischemic damage and promote functional recovery compared with vehicle pretreatment.

## Discussion

We demonstrated here that endothelial S1PR1 expression and cell proliferation were increased in ipsilateral leptomeningeal anastomoses in a model of cerebral hypoperfusion. In CCAO animals, treatment with SEW2871, a selective agonist of S1PR1, promoted better CBF recovery in the ipsilateral cerebral cortex in parallel with increased vasodilation in the leptomeningeal anastomoses rather than promoting endothelial cell proliferation. SEW2871 treatment of CCAO animals reduced the infarct volume after subsequent pMCAO and showed significant improvement on elevated body swing test and neurological deficit score.

This suggests that leptomeningeal collateral growth in response to a selective agonist of S1PR1 is functionally effective in helping to preserve CBF after acute proximal intracranial artery occlusion.

### Upregulation of S1PR1 Expression on Endothelial Cells in Leptomeningeal Arteries Exposed to Shear Stress

After LtCCAO surgery, which induces shear stress stimulation of leptomeningeal anastomosis in C57Bl/6 mice [13–15], we observed a significant increase in the number of proliferating cells in the ipsilateral leptomeningeal arteries; this was most pronounced 7 days after surgery, as previously reported [18]. In parallel with the increase in S1PR1 mRNA levels, endothelial S1PR1 expression in the ipsilateral leptomeningeal arteries was significantly higher after LtCCAO than in sham mice, gradually increasing to a peak at 7 days and remaining high at 14 days. C57Bl/6 mice show significant decreases in CBF after unilateral CCAO[13, 15] because of hypoplasia of the posterior communicating arteries [19]. In this model, the decrease in CBF is significantly smaller in the ACA area than in the MCA area, thus inducing a pressure gradient between the MCA and ACA areas [13, 15]. The sudden decrease in CBF in the MCA area after unilateral CCAO increases the velocity of flow in the vessels of the leptomeningeal anastomoses connecting the high-pressure (ACA) area with the low-pressure (MCA) area, thus causing an increase in fluid shear stress on the leptomeningeal anastomoses between the MCA and ACA [6, 15]. Pronounced cell proliferation and increased S1PR1 expression on endothelial cells are observed under conditions of increased shear stress [11, 18], and expression of S1PR1 protein increases after 10 min of shear stress application to cultured human umbilical vein endothelial cells [11]. In wild-type mice, increased immunoreactivity to S1PR1 protein has been observed in the descending aorta after its exposure to high levels of shear stress [11]. Here, we first found increased S1PR1 expression in the ipsilateral leptomeningeal arteries, not on the contralateral side, in chronic cerebral hypoperfusion. Our results suggest that the difference in flow-dependent S1PR1 expression is associated with increased shear stress in leptomeningeal arteries.

Collateral artery growth is an adaptive process that occurs after artery occlusion and is triggered by shear stress [6, 18]. Shear stress activates mechanosensors on the endothelial cell surface [7, 20], and S1PR1 was recently reported as an important mechanosensor on endothelial cells, which respond to shear stress to transduce flow-mediated signaling [11, 20, 21]. We suggest that exogenous activation of S1PR1 during the timing of increased S1PR1 expression stimulates collateral growth under increased shear stress conditions.

### S1PR1 by Selective Agonist Promotes Leptomeningeal Collateral Growth under Shear Stress Conditions

S1PR1 is an important mechanosensor on endothelial cells, which respond to shear stress to transduce flow-mediated signaling, including that of the Akt/eNOS (vasodilation-related) and ERK1/2 (cell proliferation–related) pathways [11, 20, 21]. SEW2871 is a highly selective S1PR1 agonist that activates S1PR1 [22]. Activating S1PR1 may enhance vasodilation in collateral arteries in the brain by activating eNOS, thus promoting increased production of NO [12], which regulates vascular tone [23]. Our exogenous SEW2871 administration significantly improved CBF recovery in the ipsilateral cerebral cortex between the MCA and ACA areas, increased eNOS phosphorylation in leptomeningeal arteries, and increased the number and diameter of leptomeningeal anastomoses after LtCCAO. There were no differences in the number of Ki-67-positive mitotic cells in the ipsilateral leptomeningeal arteries 14 days after LtCCAO between SEW group and vehicle group. Therefore, the Akt/eNOS vasodilation pathway may be more important than cell proliferation pathway in S1PR1 selective agonist–induced collateral growth of the leptomeningeal arteries. In a previous study in a cardiopulmonary bypass rat model, in which shear stress is activated by the change in arterial pressure [24], SEW2871 enhanced coronary artery dilation by activating S1PR1 on endothelial cells, which release NO through activation of eNOS [25]. However, SEW2871 has no significant vasodilation effect on isolated rat arteries in the absence of shear stress [23]. SEW treatment did not enhance collateral growth without CCAO surgery in our experiment (Fig 5C). Given this information, the vasodilatory action of S1PR1-selective agonists requires shear stress. Endothelial S1PR1 activation is also reported to enhance cell-to-cell adhesion and promote vascular stability [10]. In accordance with these studies, persistent effect on dilating ipsilateral leptomeningeal arteries of SEW pretreated animals before or even after pMCAO (Figs 5B, 5C and 6B), might be due to the vasodilation effect by eNOS-derived NO production and the S1PR1-dependent vascular stabilization. Knockout of S1PR1 in mice is embryonically lethal [26]; we administered VPC23019, an S1PR1 inverse agonist to S1PR1 to assess the vasodilating function of modulating S1PR1 [27]. Enhancement of collateral growth was inhibited by the co-administration of VPC23019; therefore, the enhancement was most likely mediated by S1PR1 activation.

### Improvement of Neurologic Function and Reduction of Infarct Volume after pMCAO in S1PR1 Agonist–treated Animals

S1PR1-selective agonist treatment reduced the infarct volume and improved neurological function after subsequent pMCAO. A previous study revealed that the neuroprotective effects of SEW2871 and the non-selective S1PR1 agonist FTY720 against cerebral ischemia in a mouse stroke model occurred by means of apoptosis prevention [28] and a reduction in infiltration by immune cells [29]. However, we believe that the enhanced collateral growth of leptomeningeal anastomoses in response to S1PR1-selective agonist administration was the most important mechanism behind the protective effect in our study, given that protection by enhancement of collateral growth is provided by hypoperfusion surgery [13] or granulocyte macrophage colony-stimulating factor administration [14] before pMCAO.

FTY720 induces receptor internalization and inactivation on lymphocytes [30], whereas SEW2871 is specific to and does not inactivate S1PR1 [22, 25]. It also has less of an effect than FTY720 on the number of circulating lymphocytes [25]. SEW2871 has a short half-life *in vivo*: phosphorylation of Akt peaks 1 h after SEW2871 administration [22, 25] and returns to baseline in 4.5 h [22]. Unlike in other studies, in which an S1PR1 agonist has been administered immediately before or after (or both before and after) MCAO surgery [28, 29], we terminated the administration of SEW2871 seven days before subsequent pMCAO. Therefore, the effects of SEW2871 in preventing neuronal death [28] or reducing lymphocyte infiltration for ischemic brain [29], as mentioned in other studies, may have contributed minimally in our model. S1PR1 was previously reported to be up-regulated in neurons in focal ischemic mice model and astrocytes in experimental autoimmune encephalomyelitis mice model [28, 31]. In our study, however, S1PR1 expression in those brain parenchymal cells was not increased after LtCCAO. Since S1PR1 signals from immunofluorescence staining were also increased on endothelial cells in the ipsilateral microvessels of cerebral cortex after LtCCAO (Fig 4C), SEW2871 pretreatment might enhance some functions of endothelial S1PR1 in cerebral microvessels such as promoting vascular stability [10, 11], or maintaining vascular permeability [32]. We believe that the infarct volume reduction and functional recovery in our SEW2871 pretreated group were due to leptomeningeal collateral vessel dilation and partly due to altered endothelial function in cerebral microvessels, results in CBF improvement in response to S1PR1 activation.

In conclusion, an S1PR1 selective agonist enhanced collateral growth and led to strong infarct size reduction after subsequent focal ischemia in a chronic hypoperfusion mice model. The pharmacological action of S1PR1-selective agonists in enhancing collateral growth and thus exerting protective effects is a potential therapeutic target in patients with intracranial major artery stenosis or occlusion.

## Supporting Information

S1 Appendix Raw data of qRT-PCR analysis of S1PR1 mRNA in ipsilateral cortex of sham-operated and LtCCAO animals. (XLSX)

S1 Fig SEW2871 treatment promoted dilation of ipsilateral leptomeningeal arteries and eNOS phosphorylation, but not proliferation of endothelial cells after CCAO surgery. (A) Average areas of ipsilateral leptomeningeal arteries in the vehicle, SEW, and SEW+VPC groups. (n = 6 for sham, 4 for vehicle, 4 for SEW, and 4 for SEW+VPC; ***P < 0.001 compared with vehicle and SEW+VPC group; one-way ANOVA followed by Tukey–Kramer post hoc test. (B) Average percentage of the CD31/S1PR1 double-positive area of the total CD31 positive area in ipsilateral leptomeningeal arteries 14 days after LtCCAO (left). Average numbers of Ki-67 positive cells in leptomeningeal arteries (per section) in the SEW and vehicle groups 14 days after left CCAO (right) (n = 4 for each group; by one-way ANOVA followed by Tukey–Kramer post hoc test). CCAO, common carotid artery occlusion; SEW, sphingosine-1-phosphate receptor-1 (S1PR1) selective agonist; VPC, S1PR1 inverse agonist.
(TIF)

S2 Fig SEW2871 treatment enhances collateral growth only on ipsilateral leptomeningeal arteries after unilateral CCAO. (A) Representative images of superficial vessels after SEW treatment, as assessed after latex perfusion. A magnified image of the box in the upper panel is shown in the lower panel. Arrowheads indicate leptomeningeal anastomoses between the anterior and middle cerebral arteries (bar = 200 μm). (B) Average diameters and average numbers of leptomeningeal anastomoses between the anterior and middle cerebral arteries, as assessed after latex perfusion. (n = 7 for each group; ***P < 0.001 compared with ipsilateral side; independent samples Student’s t-test). SEW, sphingosine-1-phosphate receptor-1 (S1PR1) selective agonist; VPC, S1PR1 inverse agonist.
(TIF)

S1 Table The number of included animals. (DOCX)
