# Supplementary figures and images for: Sphingosine-1-Phosphate Receptor-1 Selective Agonist Enhances Collateral Growth and Protects against Subsequent Stroke
Source: PLoS One. 2015 Sep 14;10(9):e0138029. doi: 10.1371/journal.pone.0138029 (PMC4569572; doi:10.1371/journal.pone.0138029)

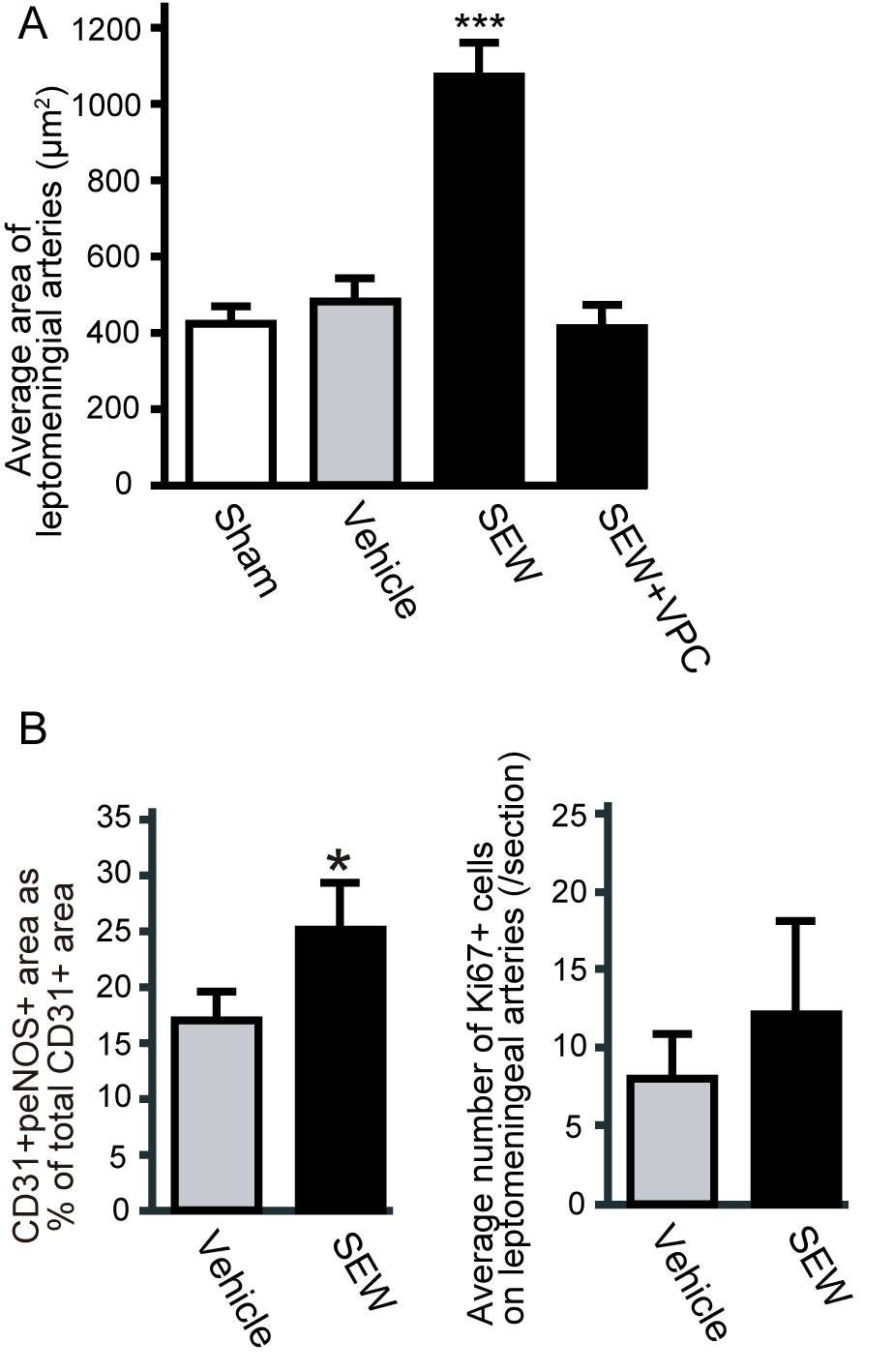

Supplement: S1 Fig — (A) Average areas of ipsilateral leptomeningeal arteries in the vehicle, SEW, and SEW+VPC groups. (n = 6 for sham, 4 for vehicle, 4 for SEW, and 4 for SEW+VPC; ***P < 0.001 compared with vehicle and SEW+VPC group; one-way ANOVA followed by Tukey–Kramer post hoc test. (B) Average percentage of the CD31/S1PR1 double-positive area of the total CD31 positive area in ipsilateral leptomeningeal arteries 14 days after LtCCAO (left). Average numbers of Ki-67 positive cells in leptomeningeal arteries (per section) in the SEW and vehicle groups 14 days after left CCAO (right) (n = 4 for each group; by one-way ANOVA followed by Tukey–Kramer post hoc test). CCAO, common carotid artery occlusion; SEW, sphingosine-1-phosphate receptor-1 (S1PR1) selective agonist; VPC, S1PR1 inverse agonist. (TIF) [file pone.0138029.s002.tif]

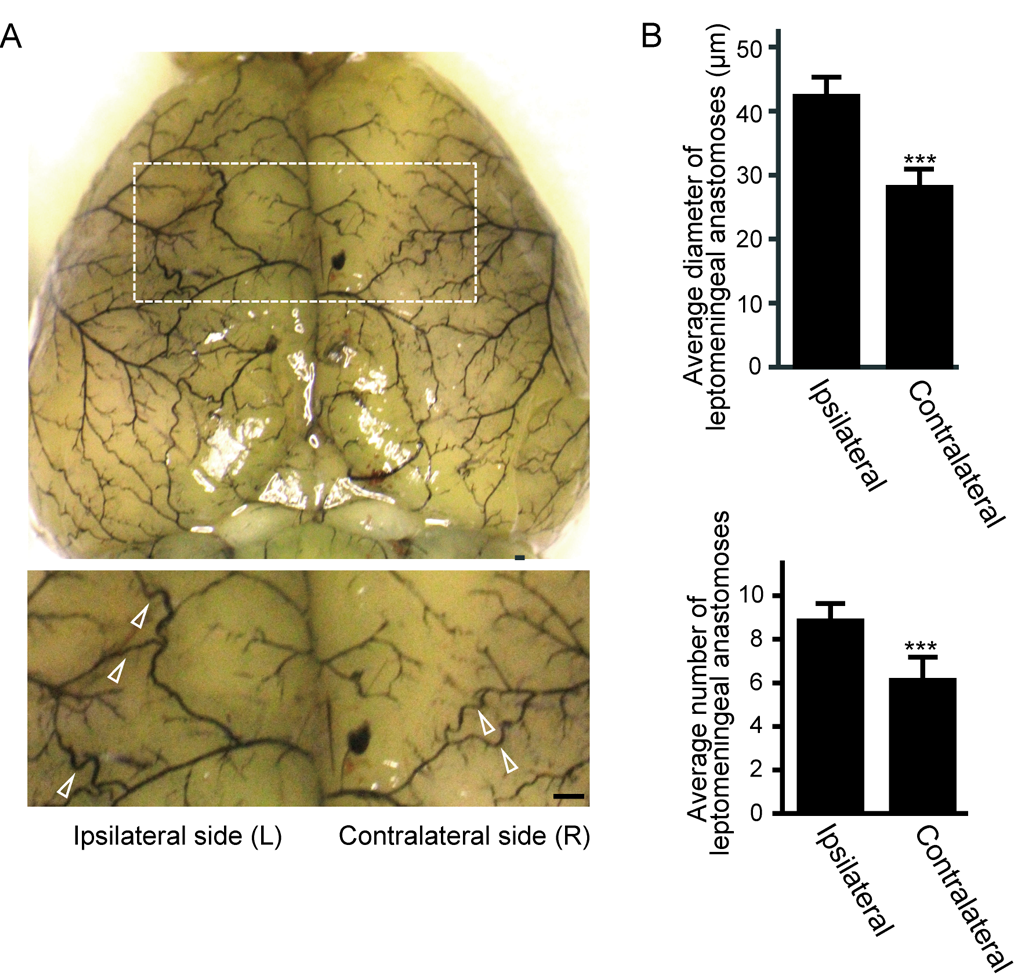

Supplement: S2 Fig — (A) Representative images of superficial vessels after SEW treatment, as assessed after latex perfusion. A magnified image of the box in the upper panel is shown in the lower panel. Arrowheads indicate leptomeningeal anastomoses between the anterior and middle cerebral arteries (bar = 200 μm). (B) Average diameters and average numbers of leptomeningeal anastomoses between the anterior and middle cerebral arteries, as assessed after latex perfusion. (n = 7 for each group; ***P < 0.001 compared with ipsilateral side; independent samples Student’s t-test). SEW, sphingosine-1-phosphate receptor-1 (S1PR1) selective agonist; VPC, S1PR1 inverse agonist. (TIF) [file pone.0138029.s003.tif]
