# Supplementary material for: Sphingosine-1-Phosphate Receptor-1 Selective Agonist Enhances Collateral Growth and Protects against Subsequent Stroke
Source: PLoS One. 2015 Sep 14;10(9):e0138029. doi: 10.1371/journal.pone.0138029 (PMC4569572; doi:10.1371/journal.pone.0138029)
Supplement: S1 Table — (DOCX) [file pone.0138029.s004.docx]

**SUPPORTING INFORMATION**

**Sphingosine-1-phosphate receptor-1 selective agonist enhances collateral growth and protects against subsequent stroke**

Masahiko Ichijo^1^, Satoru Ishibashi^1*^, Fuying Li^1^, Daishi Yui^1^, Kazunori Miki^2^, Hidehiro Mizusawa^3^, Takanori Yokota^1^

^1^ Department of Neurology and Neurological Science, Tokyo Medical and Dental University, Tokyo, Japan

^2^ Department of Endovascular Surgery, Tokyo Medical and Dental University, Tokyo, Japan

^3^ Department of Neurology, National Center of Neurology and Psychiatry, Tokyo, Japan

| **Table S1. The Number of Included Animals** | | | | | |
| --- | --- | --- | --- | --- | --- |
|  | **Latex perfusion analysis of leptomeningeal arteries after LtCCAO (Total number 14)** | | | | |
|  |  |  |  |  | n |
|  |  | Sham | | | 7 |
|  |  | LtCCAODay14 | | | 7 |
|  |  |  |  |  |  |
|  | **Histological analysis after LtCCAO (Total number 30)** | | | | |
|  |  |  |  |  | n |
|  |  | Sham | | | 6 |
|  |  | 1 day after CCAO | | | 6 |
|  |  | 4 day after CCAO | | | 6 |
|  |  | 7 day after CCAO | | | 6 |
|  |  | 14 day after CCAO | | | 6 |
|  |  |  |  |  |  |
|  | **qRT-PCR analysis after LtCCAO (Total number 14)** | | | | |
|  |  | Sham | | | 2 |
|  |  | 1 day after CCAO | | | 3 |
|  |  | 4 day after CCAO | | | 3 |
|  |  | 7 day after CCAO | | | 3 |
|  |  | 14 day after CCAO | | | 3 |
|  |  | | | | |
|  | **Pharmacological analysis of each treatment group (Total number 31)** | | | | |
|  |  |  |  |  | n |
|  |  | Sham surgery + DMSO treatment (Sham group) | | | 7 |
|  |  | LtCCAO surgery + DMSO treatment (Vehicle group) | | | 7 |
|  |  | Sham surgery + SEW treatment (SEW group without CCAO) | | | 6 |
|  |  | LtCCAO surgery + SEW treatment (SEW group) | | | 7 |
|  |  | LtCCAO surgery + SEW+VPC treatment (SEW+VPC group) | | | 4 |
|  |  |  |  |  |  |
|  | **Histological analysis of each treatment group (Total number 26)** | | | | |
|  |  |  |  |  | n |
|  |  | Sham surgery + DMSO treatment (Sham group) | | | 6 |
|  |  | LtCCAO surgery + DMSO treatment (Vehicle group) Day7 Sacrifice | | | 4 |
|  |  | LtCCAO surgery + DMSO treatment (Vehicle group) Day14 Sacrifice | | | 4 |
|  |  | LtCCAO surgery + SEW treatment (SEW group) Day7 Sacrifice | | | 4 |
|  |  | LtCCAO surgery + SEW treatment (SEW group) Day14 Sacrifice | | | 4 |
|  |  | LtCCAO surgery + SEW+VPC treatment (SEW+VPC group) | | | 4 |
|  |  |  |  |  |  |
|  | **Pharmacological analysis after subsequent pMCAO (Total number 18)** | | | | |
|  |  |  |  |  | n |
|  |  | Sham surgery + DMSO treatment (Sham group) | | | 6 |
|  |  | LtCCAO surgery + DMSO treatment (Vehicle group) | | | 6 |
|  |  | LtCCAO surgery + SEW treatment (SEW group) | | | 6 |
